# Comparison of Two Donor Liver Procurement Methods for Treatment of Pediatric Acute Liver Failure

**DOI:** 10.3389/fped.2022.816516

**Published:** 2022-03-03

**Authors:** Jiahao Pei, Conghuan Shen, Ruidong Li, Yifeng Tao, Lu Lu, Weiming Chen, Xinbao Xie, Zhengxin Wang

**Affiliations:** ^1^Department of General Surgery, Huashan Hospital of Fudan University, Shanghai, China; ^2^Department of Intensive Care Unit, Children's Hospital of Fudan University, Shanghai, China; ^3^Department of Liver Disease, Children's Hospital of Fudan University, Shanghai, China

**Keywords:** liver transplantation, living donor, donor hepatectomy, donors and donation, donor follow-up, organ procurement, pediatric acute liver failure (PALF)

## Abstract

**Background:**

To evaluate the difference and efficacy of two donor liver procurement methods for treatment of pediatric acute liver failure (PALF) by living donor liver transplantation (LDLT).

**Methods:**

A total of 17 patients (12 men, 5 women) with PALF who underwent LDLT in our hospital between October 2016 and October 2020, and prognostic efficacy of donors and recipients using two donor liver procurement methods were analyzed.

**Results:**

The donors and recipients were both divided into laparoscopic (7 cases) and open (10 cases) donor liver procurement groups. In the recipients, two deaths occurred in the laparoscopic group and one in the open group, and there were three postoperative complications in the laparoscopic group and six in the open group. The cumulative 1-year and 3-year survival rates in the laparoscopic group and the open group were 80.0% and 85.7% separately. There was no difference in the postoperative survival and complications rates between the two groups. In the donors, the operation time, postoperative hospital stay, and blood loss of the laparoscopic group was significantly reduced compared with the open group (*P* ≤ 0.01). No death or serious complication occurred in either donor group.

**Conclusion:**

Laparoscopic donor liver procurement is worth recommending than open donor liver procurement for treatment of PALF combined with LDLT in qualified transplant centers.

## Introduction

Pediatric acute liver failure (PALF) is a rapidly progressive, end-stage disease of the liver. Even with symptomatic supportive treatment for PALF, such as drugs or an artificial liver, it is sometimes difficult to save lives. Liver transplantation (LT) sometimes is the only effective treatment. Due to the rapid progression of the disease, the shortage of donor livers makes it difficult for some children to receive rapid and effective LT. It is estimated that the mortality rate of PALF on the waiting list for LT is about 20% (1). Living donor liver transplantation (LDLT) thus significantly alleviated the current situation of liver donor shortage especially for PALF patients. In recent years, with the extensive development of LDLT in China, total laparoscopic donor liver procurement in line with the minimally invasive concept and less trauma has begun to be promoted. But whether it increases the operation time, hospital stay, costs, and complications compared with traditional open surgery has attracted considerable attention. Therefore, we retrospectively analyzed patients with PALF recently admitted to our center to investigate the difference and efficacy of different donor liver procurement methods for LDLT.

## Materials and Methods

### Inclusion Criteria and Data

We collected data from 140 pediatric LT recipients from October 2016 to October 2020; 19 of whom had PALF. We reviewed and included data from 17 patients with PALF who underwent LDLT, including sex, age, weight, blood type, pediatric end-stage liver disease (PELD) score, initial symptoms, prognosis, and complications. At the same time, the donor data were included: sex, age, weight, surgical method, intra-operative blood loss, length of hospital stay, survival status, and postoperative complications.

### Surgical Methods

Donor operations were divided into laparoscopic and open access groups. In the laparoscopic group, puncture instruments with a diameter of 1 cm were placed in the left and right mid-clavicular line and left and right axillary line, respectively. The first hilum of the liver was dissected; the left hepatic artery and the left branch of the portal vein were separated; the peripheral hepatic ductile band was fully dissected, exposing the left lateral lobe; the left lateral lobe was turned to the right; the second hilum of the liver was exposed and dissected; the left branch or the left upper margin branch of the hepatic vein was isolated and marked with blue ribbon. The liver tissue, blood vessels, and bile ducts were clipped and dissected with an ultrasonic knife along the right side of the falciform ligament with the pre-resection line marked by an electro-knife. The left hepatic duct was isolated at the level of the first hilum, then the proximal end was clipped, and the distal end was dissected until the left hepatic vein remained at the second hilum. The donor liver obtained by laparoscopy cannot be cold perfused in advance, which is different from a liver donated after cardiac death. To prevent coagulation in the graft from affecting the graft quality, after heparinization, the left hepatic artery, left portal vein, and left hepatic vein were successively clipped, and the liver was divided into two parts. Then, the liver was removed from the incision above the symphysis pubis of the lower abdomen or the woman donor's original cesarean section incision with a specimen bag. Then protamine was used for deactivating heparin after the liver was detached and removed. In the open group, a subxiphoid L-shaped incision (~15 cm in length) was made, and the remaining donor liver was obtained using the same procedure as in the laparoscopic group.

All the recipient operations were performed with the piggyback technique. The left hepatic vein of the donor, portal vein of the donor, and hepatic vein of the recipient were trimmed, and the left and right hepatic veins were trimmed into a common opening. A 5-0 polydioxanone suture was used to continuously suture the opening of the left hepatic vein of the donor liver and the opening of the recipient hepatic vein. A 6-0 polydioxanone suture was used to continuously suture the bridging vessels to the portal vein of the donor and recipient. After the end of the anastomosis, the inferior vena cava and portal vein occlusion forceps were opened, respectively. The patency of each anastomotic site and the presence of leakage were examined. A 9-0MAXON suture was used to perform end-to-end anastomosis between the donor's and recipient's left hepatic artery. After the operation, B ultrasound was used to examine the portal vein, hepatic artery patency of blood flow. Choledochoenterostomy/end-to-end bile duct anastomosis was performed after the donor and recipient bile ducts were trimmed. After checking for active bleeding and placing the drainage tube, the abdomen was closed.

### Statistical Analysis

We used SPSS version 23.0 to study cumulative survival of recipients and outcomes of donors in the laparoscopic and open groups. The mean value, median value, and maximum and minimum values of continuous variables were calculated. Mann-Whitney U test and chi-square test were used to judge the difference between the two groups. *P* < 0.05 was considered to be a significant difference.

### Use of Immunosuppressants

Immunotherapy for LT recipients included a combination of steroids and tacrolimus (FK506).

Methylprednisolone was 5 mg/kg on the first day after surgery and reduced daily thereafter. Prednisone was 0.25–1 mg/kg orally on Day 8 after surgery and was stopped about 6 months after surgery. The initial dose of tacrolimus was 0.1–0.15 mg/kg/day, and the maintained plasma concentration was 8–12 ng/mL within 1 month after surgery, 7–10 ng/mL within 2–6 months after surgery, 5–8 ng/mL within 7–12 months after surgery, and 5 ng/mL 1 year after surgery and long-term thereafter.

### Ethics

This study was approved by the Institutional Review Board at the Huashan Hospital of Fudan University. Our work complies with the Declaration of Helsinki and the Declaration of Istanbul. No illegal commercial transactions were involved.

## Results

### Basic Information

Among the 17 recipients, 12 were men and 5 were women, with a mean age of 26.7 ± 7.0 months. In donors, 8 were men and 9 were female, the mean age of the donors was 31.8 ± 1.4 years, all were parents of the recipients (Table 1). The median follow-up time of donors and recipients was 35 (1–53) months.

**Table 1:** Basic information of donors in two groups.

**ID**	**Sex**	**Age (y)**	**Prepation time (d)**	**Hospital day (d)**	**Operation time (h)**	**Type**	**Blood loss (ml)**	**WIT (s)**	**Costs ($)**	**Complication**
1	M	33	5	5	3	LLLS	150	180	5,546.04	None
2	M	40	1	5	3	LLLS	150	158	4,827.76	None
3	M	28	1	5	3	LLLS	80	160	4,882.35	None
4	M	30	2	6	3	LLLS	150	200	5,808.94	None
5	M	30	2	5	3	LLLS	200	220	5,274.51	None
6	F	30	3	5	3	LLLS	150	186	6,096.94	None
7	F	33	1	6	5	LLLS	150	178	5,105.10	None
8	F	35	2	8	8	LL	200	204	5,325.65	None
9	F	26	3	15	5.75	LLS	250	212	7,886.75	Biliary stricture
10	F	24	3	7	4	LLS	150	194	4,333.65	None
11	F	40	2	10	4	LLS	200	180	5,678.90	None
12	F	28	2	7	3	LLS	200	188	4,932.24	None
13	F	27	0.5	7	6	LLS	200	168	6,475.14	None
14	M	27	1	7	4.5	LLS	250	170	4,249.25	None
15	F	27	1	7	3.5	LLS	250	164	5,575.37	None
16	M	46	1.5	12	7	LL	350	220	7,207.84	Bile leakage
17	M	36	2	7	4.5	LLS	250	200	6,007.53	None

*M/F, Male/Female; y, year; d, days; h, hour; s, second; LLLS, laparoscopic left lateral sectionectomy; LLS, left lateral sectionectomy; LL, left hemihepatectomy; WIT, warm ischemia time costs. The currency conversion rate is USD to RMB on January 7, 2022*.

### Prognosis and Survival of the Recipient

Three children (17.6%) died: two in the laparoscopic group and one in the open group, all within 1 month after surgery. The cumulative 1-year and 3-year survival rates in the laparoscopic group and the open group were 80.0% and 85.7%, respectively, there is no difference between the two groups(*P* > 0.05). The causes of death were tension pneumothorax, cerebral hernia, and primary graft failure separately. There was no difference in postoperative survival and complication rates between the laparoscopic and open groups (Table 2).

**Table 2:** Comparasion of two groups.

**Group**	**Laparoscopic**	**Open**	***P* value**
Age (y)	34 ± 32.0	21.5 ± 26.8	0.395
Weight (kg)	13.9 ± 8.1	11.7 ± 6.7	0.557
PELD	31.1 ± 8.2	34.8 ± 11.8	0.492
Recipient complication Survival	3/7 5/7	6/10 9/10	0.419 0.360
Donor age (y)	32.3 ± 6.5	31.4 ± 5.9	0.774
Prepation time (d)	2.4 ± 1.4	1.6 ± 0.7	0.131
Operation time (h)	3.3 ± 0.75	5.0 ± 1.6	0.010^*^
Hospital stay (d)	5.3 ± 0.5	8.7 ± 2.8	0.004^*^
Blood loss (ml)	147.1 ± 35.0	230 ± 53.7	0.003^*^
WIT (s)	183.1 ± 21.8	190 ± 19.3	0.505
Costs (USD)	5364 ± 476.8	5768.1 ± 1175	0.4058
Donor complication	0/7	2/10	0.331

y, year; d, days; h, hour; s, second; PELD, pediatric end-stage liver disease; WIT, warm ischemia time ^*^means significance (P < 0.05).

There were three postoperative complications in the laparoscopic group. One died of pneumothorax, one died of cerebral hernia and infection, and another patient had chylous leakage that resolved spontaneously through fasting, anti-inflammatories, and other symptomatic support treatment.

In the open group, there were six postoperative complications. Two cases of pulmonary infection were cured by standard and accurate antibiotic therapy; abdominal bleeding and acute rejection occurred in one recipient, the recipient recovered quickly after a second exploratory laparotomy for hemostasis was performed and rejection was dismissed after immunosuppressant was adjusted to MMF; one recipient had biliary complication (stricture) 2 years after surgery. He was treated with ERCP (endoscopic retrograde cholangiopancreatography) at first, but failed, and then underwent a second choledochoenterostomy a month later and recovered at last; one case of chylous leakage was cured by conservative treatment; one recipient died of liver failure.

### Donor Liver Procurement Method and Donor Prognosis

The donor liver was derived from the left lateral lobe in 15 cases, with 7 cases (46.7%) of total laparoscopic procurement and 8 cases (53.3%) of open access. Another two donor livers were derived from the left lobe of the liver, procured by open access. The mean preoperative preparation time for all 17 cases was 1.9 ± 1.1 d. In the laparoscopic group, the mean postoperative hospital stay was 5.3 ± 0.5 d, the mean operation time was 3.3 ± 0.75 h, the mean intra-operative blood loss was 147.1 ± 35.0 mL, and no donor transfusion was performed. The mean warm ischemia time (WIT) is 183.1 ± 21.8 s in the laparoscopic group and the mean costs are about $5,364 USD. In the open group, the mean postoperative hospital stay was 8.7 ± 2.8 d, the mean operation time was 5.0 ± 1.6 h, and the mean intra-operative blood loss was 230.0 ± 53.7 mL. The mean WIT is 190 ± 19.3 s in the open group and the mean costs are about $5,768.1 USD. Both the mean operation time and the mean length of hospital stay of the laparoscopic group was significantly shorter than that of the open group (*P* ≤ 0.01), and the blood loss in the laparoscopic group was less than the open group. But there was no significant difference in the WIT and costs between the two groups (Table 2). No serious complications or death occurred in the donors. There was no postoperative complication in the laparoscopic group but two in the open group (**Table 5**). One donor with postoperative biliary leakage was discharged after conservative treatment. Another donor with postoperative bile duct stenosis, presenting as obstructive jaundice, was discharged after treatment with percutaneous transhepaticcholangial drainage (PTCD). There was no difference in the incidence of complications between the two groups. The laparoscopic donors were more satisfied with their postoperative wounds than the open donors and there was no significant psychological discomfort in the two groups (Tables 2-5).

**Table 3:** Basic charateristics of PALF recipients in laparoscopic group.

**ID**	**Sex**	**Age (M)**	**Weight (kg)**	**Etiology**	**PELD**	**Symptoms**	**Complication**	**Outcome**
1	M	42	14	NBAS gene deficency	27	Jaundice	None	Alive
2	M	5	7	Metabolic	38	Jaundice, HE	Pneumothorax	Death
3	M	48	16	HEV	20	Jaundice, HE	Chylous leakage,CMV EBV	Alive
4	M	15	9	Mitochondrial defect disorders	16	Jaundice, HE	Pulmonary infection, Cerebral infarction	Death
5	M	6	8	Metabolic	43	Jaundice, HE	None	Alive
6	M	9	8	DILI	45	Jaundice, HE	None	Alive
7	F	10	9	DILI	34	Jaundice	None	Alive

*M/F, Male/Female; DILI, drug-induced liver injury; HEV, hepatitis E virus; HE, hepatic encephalopathy; EBV, epstein-barr virus; CMV, cytomegalovirus; Metabolic, inherited metabolic disease (pathology diagnosis), but the detailed type is not clear*.

**Table 4:** Basic charateristics of PALF recipients in open group.

**ID**	**Sex**	**Age (M)**	**Weight (kg)**	**Etiology**	**PELD**	**Symptoms**	**Complication**	**Outcome**
1	F	96	30	Wilson's dease	23	Fever, Jaundice	Abodominal bleeding,AR	Alive
2	M	22	15	DILI	34	Jaundice	Biliary complication	Alive
3	M	5	8	Metabolic	43	Jaundice	None	Alive
4	F	20	8	Metabolic	33	Jaundice	None	Alive
5	M	5	8	Metabolic	40	Jaundice,HE	Pulmonary infection	Alive
6	F	72	22	Indeterminate	30	Jaundice	None	Alive
7	M	6	8	DILI	57	Jaundice,HE	Chylous leakag	Alive
8	M	13	12	Indeterminate	31	Jaundice,HE	Liver failure	Death
9	M	72	26	DILI	23	Jaundice	None	Alive
10	F	7	8	Metabolic	29	Jaundice,HE	Pulmonary infection	Alive

*DILI, drug-induced liver injury; HEV, hepatitis E virus; HE, hepatic encephalopathy; EBV, epstein-barr virus; CMV, cytomegalovirus; AR, acute rejection; Metabolic, inherited metabolic disease (pathology diagnosis), but the detailed type is not clear*.

**Table 5:** Complication on the donor and recipient.

**Laparoscopic group (Recipient)**	**Open group (Recipient)**	**Laparoscopic group (Donor)**	**Open group (Donor)**
Pneumothorax (1)	Abodominal bleeding,AR (1)	None	Biliary stricture (1)
Chylous leakage,CMV EBV (1)	Pulmonary infection (2)		Bile leakage (1)
Pulmonary infection, Cerebral infarction (1)	Chylous leakage (1)		
	Liver failure (1)		
	Biliary complication (1)		

*EBV, epstein-barr virus; CMV, cytomegaloviru; AR, acute rejection*.

## Discussion

PALF is a rapidly progressive, fatal disease in which liver disorders with significant coagulation dysfunction and hepatic encephalopathy occur in the absence of known chronic liver disease. LT is the only effective treatment for most children. The high fatality rate is due to the shortage of livers in children, the difficulty in matching blood type and size, and the narrow window of time from onset to transplantation. Even by the time a suitable liver is available, the child may have developed systemic multiple organ failure or severe brain edema, with a high rate of disability and mortality after transplantation. PALF accounted for 13.5% (19/140) of pediatric LT in our center, which was basically similar to the 12.9% reported by Baliga et al. (2), but slightly lower than the 22% reported by Miolh et al. (3). In the United Network for Organ Sharing (UNOS) data, the average waiting time for ALF is 41 d, whereas our average waiting time was 1.9 ± 1.1 d because of living donor liver. Therefore, LDLT has obvious advantages in PALF (4, 5). In recent years, LDLT has become the preferred treatment for PALF (6, 7). Oh et al. reported that LDLT accounted for 94% of cases of PALF (6); it was similar with our data of 89.5% (17/19) of PALF.

With the rapid development of laparoscopic surgery and the exploration of laparoscopic donor liver procurement, which liver procurement method is better remains clear. The 1-year and 5-year survival rates of PALF recipients was reported as from 74 to 87% (3, 8, 9). The 1-year and 3-year recipient survival rates of PALF after LDLT in our center were both 82.4% (14/17), which was similar to those reported in the literature. As we concluded, there is no difference in the survival of patients between the laparoscopic and the open groups. Similarly, there is no difference in postoperative complications between the two groups of recipients. In a word, laparoscopic donor liver procurement had no effect on the postoperative survival of the recipient, nor did it increase the incidence of postoperative complications of the recipient, which is consistent with the literature (10, 11).

Although there are few empirical reports on the use of LDLT combined with total laparoscopic donor liver procurement for treatment of PALF, the minimally invasive and safe use of laparoscopic donor liver procurement is not in doubt. In 2016, our center carried out the first total laparoscopic donor liver procurement operation in East China (12). Then the laparoscopic donor liver procurement method was promoted. It should be emphasized first that the surgeons in the laparoscopic group and the open group in our study were not the same doctors. The transplant surgeon was responsible for open liver procurement, while in the laparoscopic group, there was a surgeon who was proficient in laparoscopic hepatocellular carcinoma resection (almost 300 tables per year). Moreover, it has been widely reported that the operation time of open access living donor liver procurement is shorter in many articles (13, 14). But in our experience, the operation time was significantly reduced compared with the open approach, which is our advantage and it differs from many reports. The difference may be attributed to our laparoscopic surgeons' rich experience in laparoscopic hepatocellular carcinoma resection. What is more, from our study, we can see that the hospital stay and blood loss is less in the laparoscopic group, which means less damage to the donor. Even so, there was no significant difference in WIT and costs between the two groups. This indicates that laparoscopic methods do not increase graft risks of much more WIT or costs. Retrospective studies from many cases found that the incidence of complications was as high as 40%, including bleeding, infection, incisional hernia, and biliary complications. Occasional reports of severe complications or even death are also reported (15, 16). The case fatality rate of LDLT is 0.2–0.5%, among which, fatality is most common among donors undergoing right hemihepatectomyr (17). Although total laparoscopic donor liver procurement is safe and less invasive, it is difficult to control massive bleeding during the operation, so it is sometimes necessary to convert to open surgery. If the surgeon's technical or psychological ability is not up to standard, the safety of the donor will be threatened. In our cases, no donor died, and no serious donor complications occurred. Biliary complications occurred in two donors in the open group, while no operation-related complications occurred in the laparoscopic group. There was no significant difference between the two groups, which also proved the safety of donor liver procurement in the laparoscopic group. In addition, some small details need attention. For example, we previously found that the use of a linear stapler would cause the loss of a certain length of hepatic vein, so we use the Hem-o-Lock clip instead of a linear stapler to preserve the left hepatic vein as long as possible for anastomosis between the recipient and the graft. For the safety of the donor, three clips are generally used when the left hepatic vein is severed.

**Figure 1:**
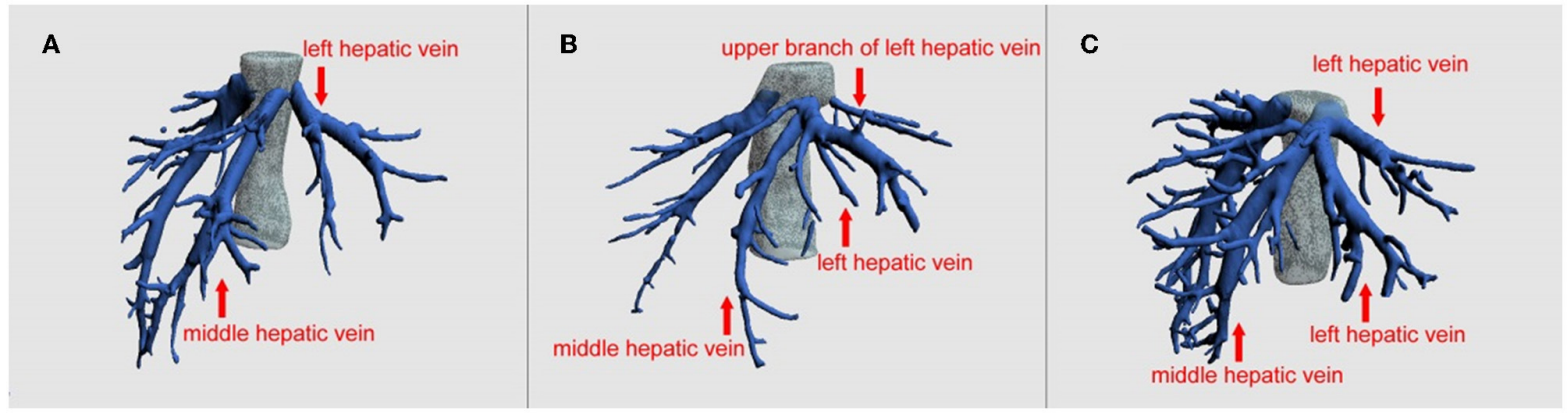
Preoperative imaging evaluation of the hepatic vein by three-dimensional reconstruction. **(A)** Direct import type (Nakamura classification Type V), **(B)** upper branch type (Nakamura classification Type IId and IIId), **(C)** indirect import type (Other types). As shown in the figure, B type and C type hepatic vein anatomy or a more complicated hepatic vein variation, for doctors without enough laparoscopic left hepatic lobe procurement experience, they should be careful to use laparoscopic surgery because it is difficult to dissociate the hepatic vein under endoscopy, it is easy to bleed, or it is difficult to obtain sufficient length for later. Regarding this aspect, our team's work has been published in the journal *Liver Transplantation* (12), and the surgical method of left hepatic vein preferential approach (LHVPA) has been proposed.

Laparoscopic donor liver procurement also has limitations. Total laparoscopic donor liver procurement requires the surgeons to be proficient in laparoscopic surgery and liver resection, and to overcome surgical space narrowing. Removal of the liver grafts from the abdominal cavity is also a very demanding procedure, which may significantly increase the WIT. But in our study, the WIT between the two groups was similar and there is also no statistical difference. Moreover, for donors with complex structures of the left hepatic vein, laparoscopic donor liver resection is not suitable. For example, donors with multiple branches of S2 and S3 hepatic vein openings have a risk of tearing and bleeding of the middle and left hepatic veins (Figure 1). Furthermore, if a right liver graft is required, total laparoscopic donor liver is difficult to obtain. One case of complete laparoscopic right lobe donor liver procurement was first reported in 2013 by Soubrane et al. (18). In our country, the Huaxi Hospital successfully completed the first adult laparoscopic right lobe donor liver procurement for LDLT in 2016 (19). In other words, experience of laparoscopic right lobe procurement is limited in our country.

What is more, in our study, the small number of cases is our limitation. We will collect more cases in the future and make efforts to conduct multi-center studies to expand the sample size.

In conclusion, despite the disadvantage above, we believe that the combination of minimally invasive laparoscopic donor liver procurement will certainly become the future direction of LDLT. We recommend LDLT combined with total laparoscopic left lobe donor liver procurement as the first option for PALF patients in qualified transplant centers.

## Data Availability Statement

The raw data supporting the conclusions of this article will be made available by the authors, without undue reservation.

## Ethics Statement

The studies involving human participants were reviewed and approved by Institutional Review Board at Huashan Hospital of Fudan University. Written informed consent to participate in this study was provided by the participants' legal guardian/next of kin. Written informed consent was obtained from the individual(s), and minor(s)' legal guardian/next of kin, for the publication of any potentially identifiable images or data included in this article.

## Author Contributions

JP: study concept and design, acquisition of data, analysis and interpretation of data, and drafting of manuscript. CS, RL, YT, LL, WC, and XX: critical revision of manuscript. ZW: critical revision of manuscript. All authors contributed to the article and approved the submitted version.

## Funding

This work was supported by National Natural Science Foundation of China (81873874 and 82071797), Clinical Research Plan of SHDC (Nos. SHDC2020CR2021B and SHDC2020CR5012).

## Conflict of Interest

The authors declare that the research was conducted in the absence of any commercial or financial relationships that could be construed as a potential conflict of interest.

## Publisher's Note

All claims expressed in this article are solely those of the authors and do not necessarily represent those of their affiliated organizations, or those of the publisher, the editors and the reviewers. Any product that may be evaluated in this article, or claim that may be made by its manufacturer, is not guaranteed or endorsed by the publisher.

## Abbreviations

LDLT: Living donor liver transplantation
PALF: Pediatric acute liver failure
PELD: Pediatric end-stage liver disease
UNOS: United Network for Organ Sharing
PTCD: Percutaneous transhepaticcholangial drainage.
